# A Conserved Leucine Zipper Motif in Gammaherpesvirus ORF52 Is Critical for Distinct Microtubule Rearrangements

**DOI:** 10.1128/JVI.00304-17

**Published:** 2017-08-10

**Authors:** Matthew S. Loftus, Nancy Verville, Dean H. Kedes

**Affiliations:** a Department of Microbiology, Immunology, and Cancer Biology, University of Virginia, Charlottesville, Virginia, USA; b Department of Medicine, Division of Infectious Diseases and International Health, University of Virginia, Charlottesville, Virginia, USA; Oregon Health & Science University

**Keywords:** HHV8, Kaposi's sarcoma-associated herpesvirus, microtubule-associated protein, RRV, rhesus monkey rhadinovirus, coiled coil, leucine zipper, tegument

## Abstract

Productive viral infection often depends on the manipulation of the cytoskeleton. Herpesviruses, including rhesus monkey rhadinovirus (RRV) and its close homolog, the oncogenic human gammaherpesvirus Kaposi's sarcoma-associated herpesvirus/human herpesvirus 8 (KSHV/HHV8), exploit microtubule (MT)-based retrograde transport to deliver their genomes to the nucleus. Subsequently, during the lytic phase of the life cycle, the maturing viral particles undergo orchestrated translocation to specialized regions within the cytoplasm, leading to tegumentation, secondary envelopment, and then egress. As a result, we hypothesized that RRV might induce changes in the cytoskeleton at both early and late stages of infection. Using confocal imaging, we found that RRV infection led to the thickening and acetylation of MTs emanating from the MT-organizing center (MTOC) shortly after viral entry and more pronounced and diffuse MT reorganization during peak stages of lytic gene expression and virion production. We subsequently identified open reading frame 52 (ORF52), a multifunctional and abundant tegument protein, as being the only virally encoded component responsible for these cytoskeletal changes. Mutational and modeling analyses indicated that an evolutionarily conserved, truncated leucine zipper motif near the N terminus as well as a strictly conserved arginine residue toward the C terminus of ORF52 play critical roles in its ability to rearrange the architecture of the MT cytoskeleton. Taken together, our findings combined with data from previous studies describing diverse roles for ORF52 suggest that it likely binds to different cellular components, thereby allowing context-dependent modulation of function.

**IMPORTANCE** A thorough understanding of the processes governing viral infection includes knowledge of how viruses manipulate their intracellular milieu, including the cytoskeleton. Altering the dynamics of actin or MT polymerization, for example, is a common strategy employed by viruses to ensure efficient entry, maturation, and egress as well as the avoidance of antiviral defenses through the sequestration of key cellular factors. We found that infection with RRV, a homolog of the human pathogen KSHV, led to perinuclear wrapping by acetylated MT bundles and identified ORF52 as the viral protein underlying these changes. Remarkably, incoming virions were able to supply sufficient ORF52 to induce MT thickening and acetylation near the MTOC, potentially aiding in the delivery viral genomes to the nucleus. Although the function of MT alterations during late stages of infection requires further study, ORF52 shares functional and structural similarities with alphaherpesvirus VP22, underscoring the evolutionary importance of MT cytoskeletal manipulations for this virus family.

## INTRODUCTION

Entry, maturation, and egress of viruses are often dependent on their ability to modulate the dynamics of the cytoskeleton (1–3). Intracellular trafficking of virions, for example, frequently takes advantage of the microtubule (MT) component of the cytoskeleton and MT-based motors for rapid and concerted transport from the periphery toward the perinuclear region. This efficient retrograde movement is particularly critical for many DNA viruses, including herpesviruses, for the nuclear delivery of their genomes as well as directed migration toward specific organelles to allow virion maturation and subsequent egress (4–7). Although Rho-GTPases control MT dynamics (8), posttranslational modification, such as the acetylation of tubulin subunits along the MT polymer, correlates with and may contribute to MT stabilization (9). Extensive stabilization can also lead to the formation of thick, cross-linked MT bundles that appear as intensely fluorescent linear structures when bound by antitubulin antibodies. These bundles are often mediated by MT-associated proteins (MAPs) (10). In light of the importance of MT-based transport in the viral life cycle, it is not surprising that a number of viruses encode proteins that stabilize microtubules (11–13), with some even acting as viral MAPs that interact directly with MTs (12, 13). Such virally induced MT stabilization thereby could promote efficient trafficking during various stages of the viral life cycle.

For the human oncogenic gammaherpesvirus Kaposi's sarcoma-associated herpesvirus (KSHV) and its close phylogenetic homolog rhesus monkey rhadinovirus (RRV), actin and microtubules play discrete roles in the uptake and nuclear delivery pathways, respectively, with the relative importance of their roles often varying by cell type (14–16). KSHV is the etiologic agent underlying Kaposi's sarcoma, primary effusion lymphoma, and multicentric Castleman's disease (17–19). KSHV adopts a primarily latent infection, but even with treatment with phorbol esters or histone deacetylase (HDAC) inhibitors, it results in only low titers in culture (20). In contrast, RRV efficiently infects rhesus monkey fibroblasts (RhFs), enters the lytic (productive) phase, and replicates to relatively high viral titers (21). This robust lytic growth in culture as well as the high degrees of sequence homology between KSHV and RRV (22, 23) make RRV a useful model to study lytic replication, including assessing the impact of individual viral gene products on infection.

In our previous work characterizing purified virions, we found that RRV *orf52* encodes a highly abundant structural tegument protein that is associated closely with the capsid (24). We subsequently demonstrated that during lytic RRV infection, open reading frame 52 (ORF52) is critical for proper tegumentation, transport of the tegument protein ORF45 out of the nucleus, as well as secondary envelopment of the maturing particle (25). ORF52 is a late lytic protein of 139 amino acids (26) with orthologs in all known gammaherpesviruses, including murine gammaherpesvirus 68 (MHV-68) (27), Epstein-Barr virus (EBV) (28), and KSHV (29, 30). The orthologs show a high degree of identity/similarity and have five strictly conserved amino acids (31). Of particular note, a previous study determined the crystal structure of a C-terminally truncated version of MHV-68 ORF52, which has a full-length primary sequence that is 63% similar to that of RRV ORF52 and is comprised of three sequential alpha helices followed by a short beta sheet (31). Transfection experiments demonstrate that the protein, at a minimum, homodimerizes within the cell, and the crystal structure suggests that it forms asymmetric, antiparallel homodimers with the added potential to form antiparallel dimer-dimer homotetramers (31). Remarkably, the structure of MHV-68 ORF52 is highly reminiscent of that of a portion of the microtubule-interacting alphaherpesvirus protein VP22 of herpes simplex virus 1 (HSV-1), despite the lack of similarity in their primary sequences (31, 32).

In light of this structural similarity to VP22, we asked whether RRV ORF52 might affect the cytoskeleton, focusing initially on MT dynamics at late stages of infection, when its expression level is highest (26). We also hypothesized that the abundance of ORF52 within the virion itself might be sufficient to affect MT dynamics during the earliest stages of infection, with the assumption that it could be released into the cytoplasm following the fusion of the viral envelope with the cellular membrane or endocytic vesicles (33). Our results indicated that RRV ORF52 had profound effects on the MT cytoskeleton. During lytic infection, ORF52 induced MT bundling and acetylation at later stages of infection, while ORF52 knockdown reversed these effects. Furthermore, we found that the transfection of ORF52 in the absence of other viral proteins was sufficient to recapitulate these MT changes and additionally led to the failure of cytokinesis and the accumulation of multinucleated cells. We report these findings along with the identification of possible key structural elements in ORF52 that appear critical for its effect on the MT cytoskeleton.

## RESULTS

### RRV infection leads to MT bundling that colocalizes with ORF52.

**FIG 1:**
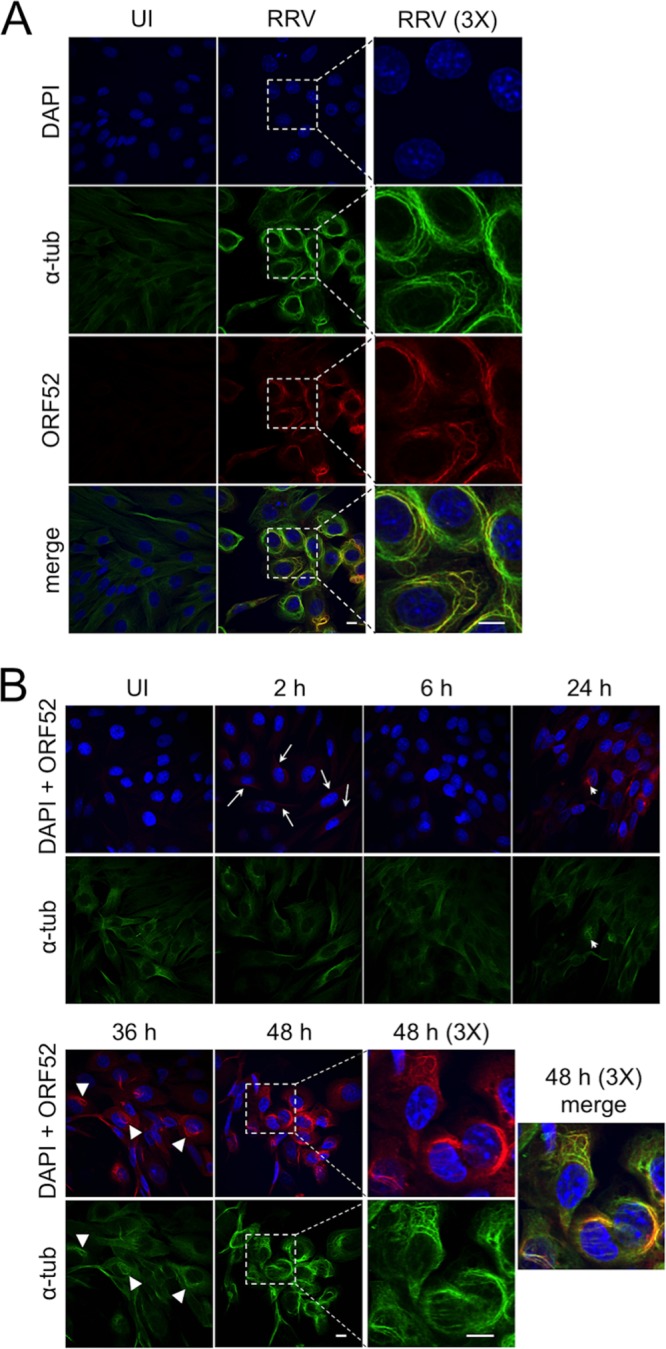
RRV infection at a low MOI leads to MT bundling, which colocalizes with ORF52. (A) hTERT-RhF cells were mock infected (uninfected [UI]) or infected with RRV (MOI = 5) for 48 h, followed by fixation and staining for DNA (DAPI) (blue), α-tubulin (α-tub) (green), and ORF52 (red), and then imaged by confocal fluorescence microscopy. The bottom images represent the merging of the images from the three stains. The third column of images shows ×3 magnifications of the regions indicated by the dashed boxes in the adjacent images. (B) hTERT-RhF cells were infected with RRV (MOI = 5), fixed at the indicated times from 2 to 48 h p.i., and stained as described above for panel A. The top rows of images represent a merge of DAPI and ORF52 staining, and the bottom rows represent α-tubulin staining. Cells within the boxed region from the 48-h time point are shown at a ×3 magnification in the adjacent column, and the final image depicts all 3 signals merged. Arrows in the images at 2 h p.i. indicate examples of cells with low levels of ORF52 likely released from the initial RRV inoculum. Thin arrowheads in the images for 24 h p.i. indicate what appears to be an MT aster, and triangular arrowheads indicate moderate MT bundling at 36 h p.i.

To assess the potential effect of RRV on MTs, we first infected telomerase-immortalized rhesus fibroblasts (hTERT-RhFs) with RRV at a multiplicity of infection (MOI) of 5 for 48 h and then detected α-tubulin by immunofluorescence (IF) microscopy. Compared to uninfected controls, infected hTERT-RhF cells showed clear evidence of bundled MTs that frequently ringed the nucleus (Fig. 1A, rows 1 and 2). To determine the localization of RRV ORF52, we costained the cells with anti-ORF52 antibodies and found a colocalization pattern that was nearly indistinguishable from that of the MT bundles (Fig. 1A, rows 3 and 4).

We next examined the temporal relationship between ORF52 expression and MT bundling following RRV infection, predicting that if ORF52 was playing a causal role, it would appear either before or at least concurrent with changes in the tubulin cytoskeleton. IF analysis revealed low levels of perinuclear accumulation of ORF52 by as early as 2 h postinfection (p.i.), but this staining was no longer evident by 6 h p.i., and we found no evidence of MT bundling at either time point (Fig. 1B). We reasoned that the early, transient accumulation of low levels of ORF52 most likely represented ORF52 associated with incoming virions and that by 6 h p.i., it was either degraded or too disperse to be detected by IF microscopy. However, by 24 h p.i., moderate levels of ORF52 reappeared, consistent with the pattern of late lytic gene expression characteristic of *de novo* RRV infection in culture (26). Although we noted no widespread MT bundling even at this time point, a subset of the RRV-infected cells demonstrated restricted bundling with the formation of MT aster-like structures that colocalized with ORF52 (Fig. 1B, arrows). These asters were essentially absent from uninfected cells. As infection progressed, ORF52 levels continued to rise, clearly colocalizing with MT bundles and reaching a peak 48 h p.i. (Fig. 1B).

**FIG 2:**
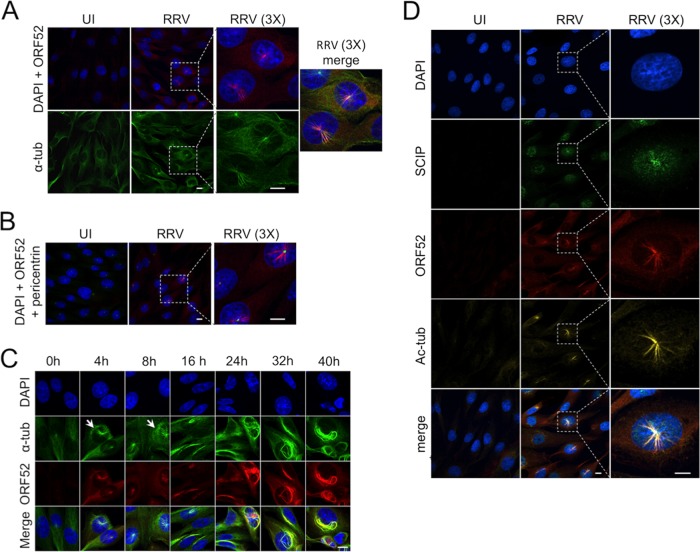
RRV infection at a high MOI leads to the transient appearance of ORF52-decorated MT asters at the MTOC and centripetal capsid accumulation. (A) Mock- or RRV-infected (MOI = 50) hTERT-RhF cells were incubated for 4 h and then stained and imaged as described in the legend of Fig. 1. The third column shows magnified (×3) views of the boxed regions in the adjacent images, and the third row represents the merging of all three stains. (B) Parallel aliquots of infected hTERT-RhF cells were stained for pericentrin (green) in addition to DAPI and ORF52. (C) Additional hTERT-RhF cells were infected at a high MOI and incubated for the times indicated. Cells were then fixed, stained, and imaged as described above for panel A. The bottom row is a merge of DAPI, ORF52, and α-tubulin. Short arrows indicate examples of cells with MT asters. (D) Mock-infected (UI) or RRV-infected (MOI = 50) hTERT-RhF cells were incubated for 4 h and then stained with DAPI, SCIP (ORF65), or ORF52. The third column shows magnified (×3) views of the boxed regions in the adjacent images, and the bottom row represents the merging of all three stains. White bars represent 10 μm.

Since MT aster formation was evident at concentrations of ORF52 that were lower than what appeared to be necessary for MT bundling, we predicted that if we infected cells at a sufficiently high MOI, ORF52 potentially released from the tegument of incoming virions might accelerate aster formation, even prior to significant levels of *de novo* late lytic gene expression (26). To test this, we infected hTERT-RhF cells at an MOI of 50 (rather than 5) and then imaged cells at 4 h p.i. We found marked aster formation in infected cells compared to uninfected controls and that ORF52 preferentially localized to these structures, which were reminiscent of microtubule-organizing centers (MTOCs) (Fig. 2A). Staining with pericentrin antibodies confirmed that the asters, which also demonstrated thickened MTs consistent with bundling, emanated from the MTOCs (Fig. 2B). A detailed time course of high-MOI infection revealed that these ORF52-decorated asters reached their peak at approximately 8 h p.i. but then began to disappear by 16 h p.i. (Fig. 2C). As with the lower-MOI infections, subsequent (24 to 40 h p.i.) and more extensive bundling appeared independent of the MTOCs and coincided with the markedly higher levels of ORF52 that arose during the peaks of late lytic gene expression. Further supporting our hypothesis that the formation of the asters reflected the effects of ORF52 released from incoming virions (4–7, 34), we also found that SCIP (ORF65) accumulated centripetally along aster-associated MTs by as early as 4 h p.i. (Fig. 2D).

### Increasing levels of intracellular ORF52 correlate and colocalize with acetylated MT bundles.

**FIG 3:**
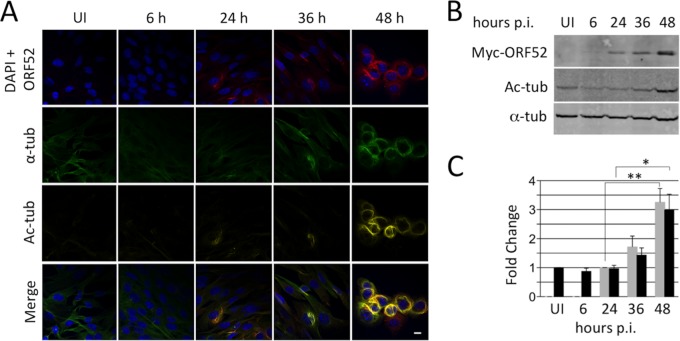
Acetylation of RRV-induced MT bundles correlates and colocalizes with ORF52 expression. (A) hTERT-RhF cells were mock infected (UI) or infected with RRV (MOI = 5) and fixed at indicated times p.i. Cells were stained for DNA (DAPI) (blue), ORF52 (red), α-tubulin (green), or acetylated tubulin (Ac-tub) (dark yellow), with the bottom row displaying merged images. (B) RRV-infected hTERT-RhF cells were collected at increasing times p.i. and analyzed for levels of ORF52 expression, tubulin acetylation, and total α-tubulin (loading control) by immunoblotting with the indicated antibodies. (C) Graphic representation of ORF52 expression (gray bars) and tubulin acetylation (black bars) from 3 biological replicates of the experiment shown in panel B. Values represent means ± standard errors of the means, normalized to the α-tubulin level for each lane. The ORF52 expression level is set to 1.0 at 24 h, and that for acetylated tubulin is set to 1.0 for uninfected cells (*, *P* < 0.05; **, *P* < 0.01 [by 2-tailed Student's *t* test]). All micrographs in panel A are at the same magnification, and the white bar represents 10 μm.

Tubulin acetylation often accompanies MT bundling and stability. To test whether RRV-induced MT bundling was likewise associated with acetylation, we infected hTERT-RhF cells with RRV at an MOI of 5 and then monitored the levels and localization of ORF52, α-tubulin, and acetylated tubulin by IF microscopy over time (Fig. 3A). The results demonstrated that the ORF52-decorated MT bundles were also acetylated and that MT acetylation appeared to be restricted to these bundles (Fig. 3A). To more quantitatively assess the relationship between ORF52 expression and acetylated tubulin, we also analyzed, in parallel, aliquots of infected cells by immunoblotting (Fig. 3B). As infection progressed, both ORF52 and acetylated tubulin levels increased, but we noted that acetylation lagged behind the increase in ORF52 levels by approximately 12 h. Figure 3C depicts the results from three separate immunoblotting experiments and underscores that acetylation followed the increase in ORF52 levels and rose approximately 3-fold over baseline by 48 h p.i. Although acetylation seemed to track well with bundled MTs in individual cells by IF microscopy even by as early as 24 h p.i., it reached statistical significance in culture by immunoblotting only at 48 h p.i.

### ORF52 is necessary for MT bundling during infection.

**FIG 4:**
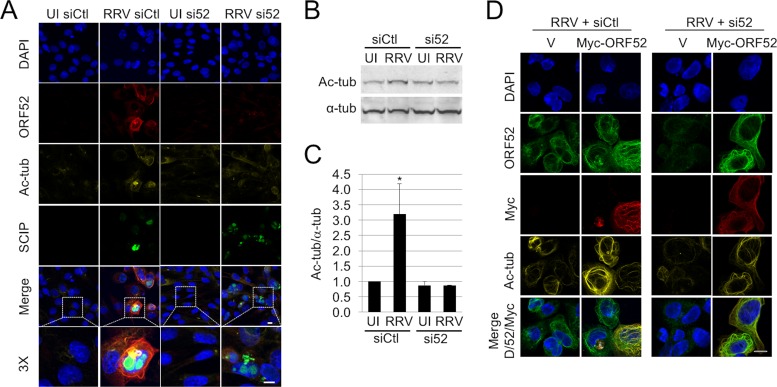
ORF52 is necessary for MT bundling during infection. (A) hTERT-RhF cells were transfected with siCtl or si52 and incubated for 24 h. Cells were then either mock infected (UI) or infected with RRV (MOI = 5); incubated for an additional 48 h; and then stained for DNA (blue), ORF65 (SCIP) (green), ORF52 (red), and acetylated tubulin (dark yellow). The penultimate row is a merge of all four colors in the column. The last row is a magnified (×3) view of the cells within the boxed area of the adjacent merged image. (B) Immunoblot analysis of the expression of acetylated tubulin and α-tubulin of hTERT-RhF cells treated the same way as described above for panel A. (Of note, the second lane, siCtl RRV, was underloaded.) (C) Graphic representation of the ratios of acetylated to alpha tubulin under the indicated conditions. The bars are the means ± standard errors of the means of data from three experiments, and the asterisks indicate ratios that differed significantly (*P* < 0.05, by Student's *t* test) in RRV-infected cells treated with control siRNA. (D) RRV-infected cells pretreated with siCtl or si52 were transfected with either the vector (V) or a plasmid encoding si52-resistant ORF52, as indicated. Forty hours later, the cells were fixed and stained as indicated. White bars represent 10 μm in panel A. siCtl, control siRNA; si52, siRNA against ORF52.

Although ORF52 colocalized to MT bundles and its expression appeared to precede their formation and acetylation, it was possible that another coexpressed RRV-encoded protein might have been responsible for this phenotype. To more directly assess whether ORF52 was necessary for RRV-induced acetylated MT bundle formation, we infected hTERT-RhF cells after small interfering RNA (siRNA) knockdown of ORF52, as we described previously (25). One day after transfection with control siRNA (siCtl) or ORF52-targeted siRNA (si52), mock- or RRV-infected hTERT-RhF cells were incubated for an additional 48 h. We then imaged the cells by IF microscopy. Pretreatment of RRV-infected cells with si52 abrogated MT bundling and acetylation compared to siCtl pretreatment (Fig. 4A). In contrast, the expression of SCIP, a capsid protein encoded by the late lytic viral gene ORF65, was evident and characteristically accumulated mainly in the nucleus (24, 25) regardless of siRNA treatment, allowing us to conclude that the lack of ORF52 expression and acetylated MT formation was not due to a block in overall RRV infection or lytic gene expression. Parallel immunoblotting buttressed these IF results, showing that the knockdown of ORF52 blocked tubulin acetylation to levels equivalent to those in uninfected hTERT-RhF cells (Fig. 4B). The average results from three independent immunoblotting experiments showed that siCtl pretreatment followed by RRV infection led to a 3-fold increase in acetylated tubulin levels by 48 h, while ORF52 knockdown blocked this effect (Fig. 4C). Finally, we were able to rescue MT bundling and acetylation in infected cells that had undergone ORF52 knockdown by subsequently transfecting the cells with an si52-resistant ORF52-expressing plasmid (Fig. 4D). Since only transfected ORF52 had a Myc tag, we were able to distinguish it from endogenous ORF52. Together, these data argued that ORF52 was necessary for MT bundling and acetylation.

### ORF52 is sufficient to cause MT bundling.

**FIG 5:**
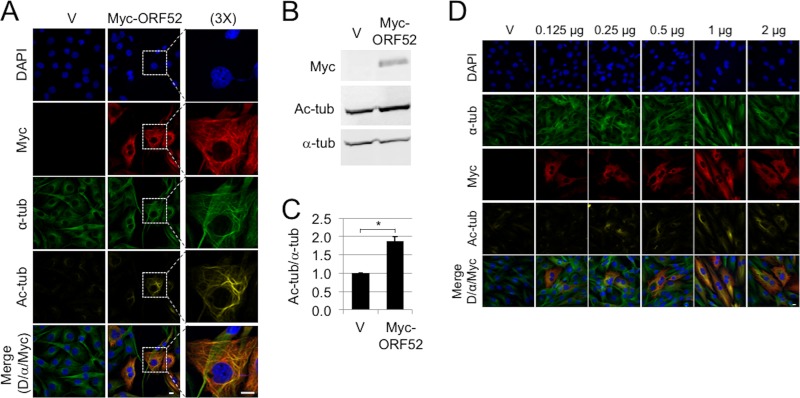
ORF52 is sufficient by itself to induce MT bundling. (A) Immortalized RhF cells were Amaxa transfected with Myc-tagged ORF52 or the empty Myc-tagged vector (V) and incubated for 48 h. Cells were fixed and stained with DAPI (blue), α-tubulin (green), acetylated tubulin (dark yellow), and Myc (red) and imaged by confocal microscopy. The last column of images shows cells from the boxed areas at a ×3 magnification. The bottom row is a merge of DAPI, ORF52, and alpha tubulin. (B) RhF cells were transfected with the vector alone or wt RRV ORF52 as described above for panel A and then analyzed 48 h later by immunoblotting with antibodies against acetylated tubulin and α-tubulin and Myc-tagged RRV ORF52 (Myc-ORF52). (C) Graphic representation of data from 3 replicative experiments depicted in panel B, quantifying the ratio of acetylated to total α-tubulin. Values were normalized to the value with the vector alone (pK-Myc). Asterisks indicate statistical significance (*P* < 0.05) determined by 2-tailed Student's *t* test. Error bars represent standard errors of the means. (D) Smaller amounts of transfected DNA lead to less obvious MT acetylation and bundling, but the failure of cytokinesis persists. hTERT-RhF cells were transfected with the empty Myc-tagged vector or increasing amounts of the Myc-ORF52-encoding plasmid as indicated. After 48 h, cells were fixed, stained, and imaged. The bottom row is a merge of DAPI, ORF52, and α-tubulin. The white bars represent 10 μm.

We next asked whether ORF52 expression alone, in the absence of other viral proteins, was sufficient to induce these changes in MTs by transfecting hTERT-RhF cells with either the empty vector pK-Myc (V) or pK-Myc-ORF52 (Myc-ORF52), which expressed the Myc-ORF52 fusion protein. Forty-eight hours after transfection, IF microscopy revealed strong Myc-ORF52 staining in a subset of cells that consistently colocalized with acetylated MT bundling (Fig. 5A). Neighboring cells within the same well that did not express Myc-ORF52 resembled the vector control, lacking MT bundling. Immunoblots from cells collected in parallel with the IF analyses revealed acetylated tubulin levels that were approximately 2-fold higher than those in untransfected hTERT-RhF cells (Fig. 5B and C). Compared to the striking IF results with individual transfected cells, the magnitudes of the immunoblot measurements of ORF52-induced acetylation were attenuated due to averaging with untransfected cells. Of note, we obtained similar results following the transfection of hTERT-RhF cells with untagged ORF52 and using anti-ORF52 antibodies, helping to determine that the MT effects that we observed were not a result of an artifact introduced by the Myc tag (data not shown).

We also noted that the distributions of acetylated and bundled MTs were different between RRV-infected and ORF52-transfected cells, with the former often displaying perinuclear wrapping and the latter showing a more widespread and reticular pattern throughout the cytoplasm. To ensure that this difference was not due to overexpression following transfection, we titrated the amount of the Myc-ORF52 expression plasmid 16-fold (Fig. 5D). Instead of recapitulating the wrapping pattern evident during RRV infection, the transfection of smaller amounts of the ORF52 plasmid served only to diminish detectable MT bundling while also decreasing evidence of acetylation. However, we note that even at the smallest amount of the ORF52 plasmid that we tested (16-fold lower than our standard amount of 2 μg/well), binucleated cells persisted despite our being unable to detect either MT acetylation or bundling at levels above those of the vector-alone control (Fig. 5D, second column). We speculate that the pattern of perinuclear MT wrapping during RRV infection (48 h p.i.) may instead reflect the dissociation of the MTOC at late stages of lytic replication rather than any significant difference in the level of ORF52 expression compared to that in transfected cells.

### Conserved residues of RRV ORF52 are important for MT bundling and acetylation.

**FIG 6:**
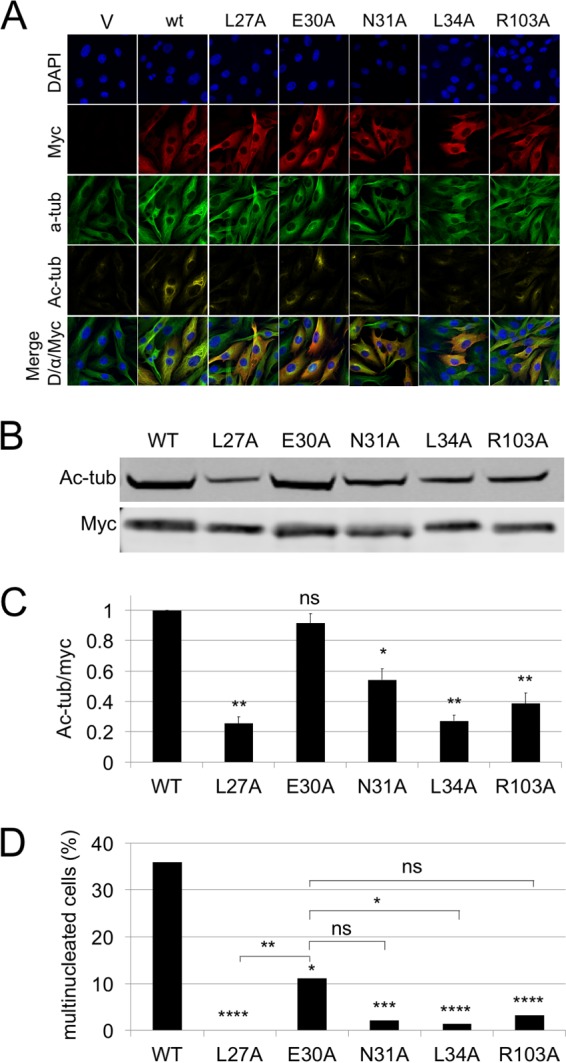
Four of the five strictly conserved residues of RRV ORF52 are important for MT bundling and acetylation. (A) hTERT-RhF cells were transfected with the pK-Myc vector, the RRV Myc-ORF52 wt, or a RRV Myc-ORF52 alanine mutant. Cells were stained with DAPI or the indicated monoclonal antibodies. The white bar indicates 10 μm. (B) hTERT-RhF cells were transfected in the same way as described above for panel A. Cells were incubated for 48 h, and cell lysates were immunoblotted with anti-acetylated tubulin and anti-Myc (Myc) antibodies. (C) Graphic representation of data from four replicative experiments depicted in panel B, quantifying the ratio of acetylated tubulin to total Myc expression. (D) Fifty to one hundred cells in randomly selected microscopy fields under each of the transfection conditions indicated in panel A were scored for single or multiple nuclei. Asterisks directly over bars in panel C indicate statistical significance determined by Student's *t* test, comparing the wt to the indicated mutant, and error bars represent standard errors of the means. For panel D, asterisks above bars indicate statistical significance determined by a chi-squared test, comparing the wt to the indicated mutant, and those above brackets are for comparisons of the E30A mutant with the other indicated mutants. *, **, ***, and **** represent *P* values of <0.05, 0.01, 0.001, and 0.0001, respectively. ns, not statistically significant.

A previously determined crystal structure of MHV-68 ORF52 (31) and protein structure prediction software (35–38) suggested that RRV ORF52 and other gammaherpesvirus orthologs likely share similar overall structures. Furthermore, sequence comparisons of these orthologs among gammaherpesviruses demonstrated five strictly conserved residues that include four residues within the predicted N-terminal α1-helix (L27, E30, N31, and L34) and a fifth residue on the β1-sheet (R103). To determine if any of these residues were critical for RRV ORF52-induced MT effects, we introduced alanine substitutions at each of these five conserved positions and expressed the mutant proteins from the pK-Myc vector, assessing transfected cells for MT bundling and acetylation. Using IF analysis, we found that the L27A and L34A mutations led to complete losses of bundling and acetylation of MTs (Fig. 6A). Although not as extreme as the two leucine mutations, the N31A and R103A mutants also showed a reduction in the ability of ORF52 to bundle or acetylate MTs, while the E30A mutant appeared to have an intermediate phenotype (Fig. 6A). To quantify the effects on acetylation, we immunoblotted aliquots of the same cells, probing for acetylated tubulin and Myc. In comparisons of the acetylated tubulin levels for each mutant, we normalized to the amount of the Myc signal under each condition, thereby accounting for variations in overall expression levels among the different constructs for each experiment (Fig. 6B). Of note, the variation in the transfection efficiencies (percent cells transfected) of all the ORF52 constructs (wild type [wt] or mutant) was minimal (mean transfection level of 32% ± 4%). Figure 6C shows a graphic display of the averages from four separate experiments, and the results approximate the data from the IF images, with the two leucine mutations showing the most pronounced loss of acetylation, followed by the R103A and N31A mutations. Even though IF analysis of individual cells transfected with the E30A mutant rather than wt ORF52 suggested lower levels of MT acetylation, the immunoblot results did not reach statistical significance.

We also noted that ORF52 expression following transfection consistently led to the accumulation of cells with two (and sometimes more) nuclei (Fig. 6A) and reasoned that this likely reflected a cytokinesis failure from perturbations in MT dynamics during mitosis. Thus, we predicted that the ORF52 mutants with an absent or attenuated ability to bundle and acetylate MTs might more closely resemble untransfected cells with intact cytokinesis and single nucleated cells. To test this expectation, we determined the fraction of transfected cells with two or more nuclei 48 h after transfection of hTERT-RhF cells with the empty vector, wt ORF52, or one of the five mutated ORF52s. We found that among cells expressing wt ORF52, just over 35% were multinucleated (Fig. 6D). In contrast, the L27A, N31A, L34A, and R103A mutants showed 0, 1.4, 2.2, and 3.2% multinucleated cells, respectively. The ORF52 E30A mutation that retained intermediate levels of MT bundling and acetylation by IF analysis and statistically indistinguishable levels of acetylation by immunoblotting led to a moderate (11%) proportion of multinucleated cells (Fig. 6D). Thus, the fraction of multinucleated cells reflected the IF results more closely and may be a more sensitive indicator of perturbations in MT function in the setting of transfection. Of note, binucleated cells were absent from RRV-infected cells, despite similarly high levels of endogenous ORF52 expression, likely due to virally induced cell cycle arrest (39, 40).

**FIG 7:**
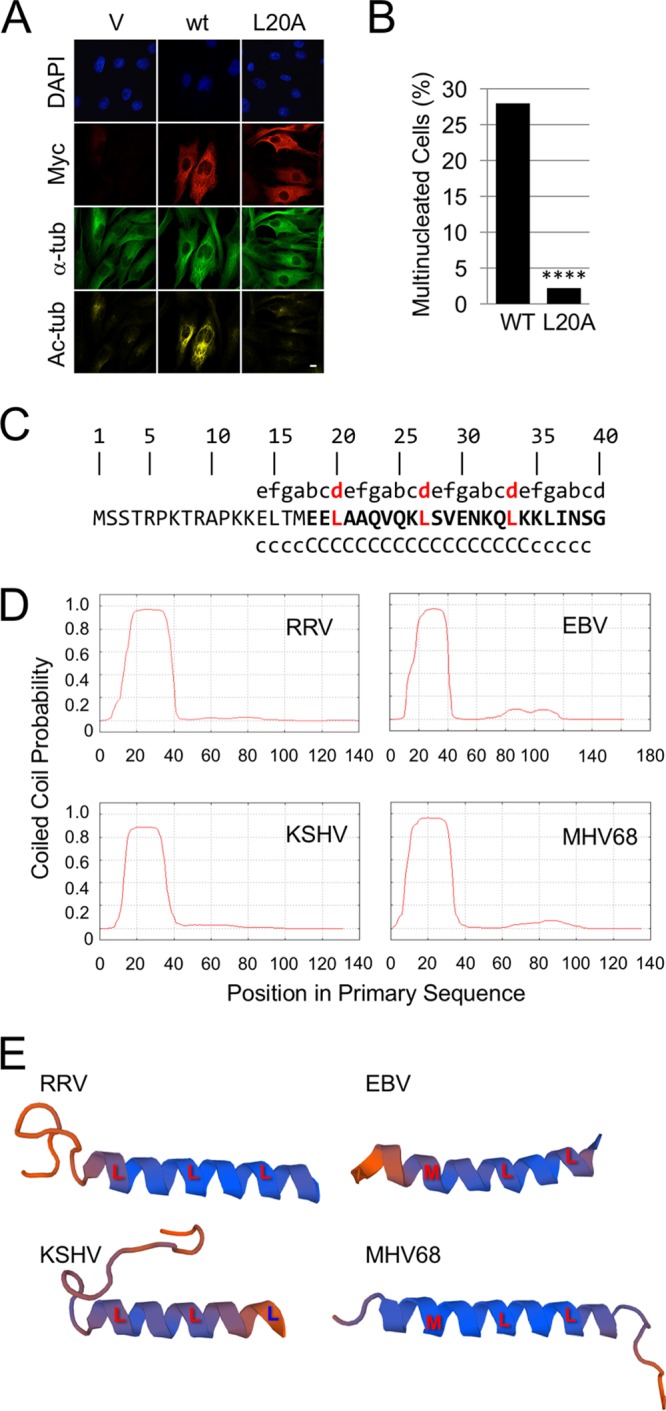
The N-terminal region of RRV ORF52 contains a single, evolutionarily conserved heptad repeat suggestive of a truncated leucine zipper that was essential for MT bundling and acetylation. (A) IF analysis of hTERT-RhF cells 48 h after transfection with the pK-Myc vector (V), the RRV ORF52 wt (wt), or the RRV ORF52 L20A mutant. (B) Multinucleated cells were quantified as described in the legend of Fig. 5, with asterisks indicating a *P* value of <0.0001 as determined by a chi-squared test. (C) The first 40 amino acids of RRV ORF52. The top line represents heptad repeat positions a through g, with the critical d position shown in red. The second line is the amino acid sequence, with bold font indicating the predicted α-helix (37, 38, 42). The third line delineates the coiled-coil prediction, with c and C representing coiled-coil probabilities of >50% and >90%, respectively, using the Marcoil algorithm (37). (D) Profiles of coiled-coil predictions for RRV, KSHV, EBV, and MHV-68 ORF52 orthologs over the length of each protein, showing positional conservation. (E) Swiss-Model-predicted three-dimensional structures of the first 40 amino acids of the RRV, EBV, KSHV, and MHV-68 ORF52 orthologs, using the MHV-68 ORF52 crystal structure as the template and showing the three, heptad d-position amino acids (L or M) aligning along the same side of the α-helix.

Since the two ORF52 mutations with the most pronounced loss of MT bundling and acetylation were L27A and L34A, we asked whether their heptad separation might also be important, noting that the residue at position 20 is also a leucine. Although not perfectly conserved, a third leucine is present in this relative position in most gammaherpesvirus ORF52 orthologs, and when it is not present, the residue at this position is replaced by either a methionine or an isoleucine residue, the two alternative amino acids that still favor the coiled-coil structure of leucine zipper domains (41). To more directly assess whether Leu20, -27, and -34 represented the boundaries of a truncated but still possibly functional leucine zipper, we used IF microscopy to analyze the effect of the ORF52 L20A mutant on MT bundling as we had done for the other mutants. We found that the ORF52 L20A mutant, like L27A and L34A, also abrogated MT bundling and acetylation (Fig. 7A) while also reversing the generation of multinucleated cells evident with wt ORF52 expression (Fig. 7B). Thus, an alanine substitution at any one of these three positions was sufficient to disrupt the MT phenotype. Together, these data strongly suggested that the heptad repeat and, therefore, most likely a coiled-coil motif in this N-terminal portion of the protein likely played a role in inducing the MT effects.

Using the Marcoil coiled-coil prediction algorithm (37, 42) to analyze the primary RRV ORF52 sequence, we noted that the polypeptide encompassing residues 14 to 39 and containing the heptad repeats had a 50 to 97% probability of forming a coiled coil (Fig. 7C). Comparison of the RRV ORF52 sequence with the crystal structure of MHV-68 ORF52 demonstrated that this region lies within the first of three sequential alpha helices (31). Of note, the Marcoil algorithm generated a nearly identical coiled-coil probability profile for a wide range of gammaherpesvirus ORF52 orthologs (Table 1), including those of KSHV, EBV, and MHV-68 (Fig. 7D). Analysis of these orthologs with a second coiled-coil prediction algorithm, PCOIL (43), gave qualitatively similar results in the homologous regions of the proteins (Table 1). Three-dimensional modeling of the first 40 amino acids of ORF52 in RRV, KSHV, EBV, and MHV-68, based on the MHV-68 ORF52 crystal structure template (35, 36, 38), demonstrated positional conservation of the heptad hydrophobic residues. The ORF52 α1-helix of each of the viruses had these hydrophobic residues on the same face and, furthermore, a leucine in each of the fourth or “d” positions of the heptad repeats except for a methionine substitution in the most N-terminal d positions, residues 22 and 13, in EBV and MHV-68, respectively (Fig. 7E).

**TABLE 1:** Positions and lengths of coiled-coil N-terminal segments at a minimum probability of 0.50 from 10 gammaherpesvirus ORF52 orthologs

ORF52 ortholog	GenBank accession no.	Marcoil^*a*^		PCOIL^*b*^					
		Positions (residues)	Length (amino acids)	Window length of 14		Window length of 21		Window length of 28	
				Positions (residues)	Length (amino acids)	Positions (residues)	Length (amino acids)	Positions (residues)	Length (amino acids)
Rhesus monkey rhadinovirus H26-95 ORF52	AAF60030.1	14–39	26	14–40	27	7–40	34	7–42	36
Human gammaherpesvirus 8 (KSHV) ORF52	ALH44822.1	14–35	22	11–37	27	9–40	32	5–41	37
Human gammaherpesvirus 4 (EBV) BLRF2	CEQ32352.1	16–40	25	17–42	26	12–42	31	11–45	35
Bovine gammaherpesvirus 4 hypothetical protein	AEL29796.1	14–37	24	12–38	27	5–41	37	5–42	38
Bovine gammaherpesvirus 6 ORF52	YP_009042031.1	14–38	25	12–38	27	9–42	34	8–42	35
Callitrichine gammaherpesvirus 3 (marmoset) ORF46	NP_733900.1	22–48	27	20–45	26	19–51	33	16–52	37
Murid gammaherpesvirus 4 (MHV-68) ORF52	NP_044889.1	9–32	24	8–33	26	7–36	30	11–42	32
Wood mouse herpesvirus virion protein G52	ACY41123.1	9–32	24	8–33	26	7–36	30	7–37	31
Equid gammaherpesvirus 2 virion protein G52	AIU39497.1	20–46	27	20–43	24	20–46	27	20–49	30
Porcine lymphotropic herpesvirus 1 (unknown)	AAM22154.1	14–38	25	12–38	27	10–42	33	5–42	38
Mean ± SD			24.9 ± 1.5		26.3 ± 0.95		32.1 ± 2.8		34.9 ± 2.9

a See reference 37.

b See reference 41.

## DISCUSSION

In this report, we demonstrate that RRV infection leads to striking circumferential “wrapping” of nuclei by acetylated MT bundles. These effects are most pronounced at late stages of infection during peak levels of virion maturation and egress (44), and the late lytic protein ORF52, which colocalizes with MT bundles, is both necessary and sufficient to approximate these pronounced changes in MT organization. These cytoskeletal changes are reminiscent of the alterations induced by the overexpression of the structurally related VP22 protein of HSV-1 (32, 45, 46) as well as the otherwise unrelated cellular centrosomal protein Cep57 (47). Surprisingly, our data also indicate an early function for virion-associated ORF52. Shortly after infection and prior to the *de novo* expression of late lytic proteins (26), incoming viral particles release most if not all of their tegument-associated ORF52 following fusion and entry. Other studies also described the release of a subset of tegument proteins upon herpesvirus entry (48, 49). The bolus of incoming ORF52, an abundant structural protein (24), appears sufficient, at higher MOIs, to induce MT aster formation at the MTOC. These asters comprise bundled and acetylated MTs that also colocalize with ORF52. Furthermore, we found that incoming capsids rapidly accumulate in the perinuclear region and are particularly concentrated at MTOC-associated asters, likely as a result of capsid retrograde transport along MTs (34).

Although recent work on adenovirus-associated virus (AAV) suggests that the intact MTOC acts as part of an innate intracellular antiviral defense mechanism (50), we propose that the opposite is likely true with herpesviruses, including RRV. Early stabilization of MTs emanating from the MTOC might facilitate efficient capsid and, therefore, genome delivery to the nucleus. Consistent with this notion, previous work with RRV, KSHV, and other herpesviruses demonstrated that a disruption of MTs and/or retrograde transport with dynein inhibitors decreases the postentry transduction efficiency (7, 34, 51). Furthermore, previous studies also showed that the binding of KSHV to its α3β1 integrin receptor leads to transient elevations of levels of Rho-GTPases that in turn promote MT acetylation and aid in the nuclear delivery of capsids to the nucleus (7, 52–55). However, these viral binding effects peak at approximately 60 min for KSHV (7) and 30 min for RRV (M. S. Loftus and D. H. Kedes, unpublished observations) and approach background levels by 3 h. Therefore, we speculate that MT binding and acetylation in the region of the MTOC from virion-released ORF52 evident at 4 h p.i. could prolong the MT stabilization effect, further enhancing viral genome delivery to the nucleus.

Importantly, we found that the ectopic expression of ORF52 leads to MT acetylation and bundling phenotypes that are reminiscent of the patterns evident during late RRV infection, indicating that no other viral protein is necessary to induce these overall cytoskeletal changes. The caveat to this conclusion, however, is that in RRV-infected cells, the ORF52-decorated MT bundles frequently assume a perinuclear, circumferential, or wrapping pattern, in contrast to the reticular pattern emanating from the MTOCs in ORF52-transfected cells. We speculate that the MTOC may be disrupted in infected cells by an additional viral cofactor(s). If this is the case, it is reasonable to assume that MT nucleation would then arise from other sites (e.g., the Golgi apparatus), thereby leading to bundled MTs with radically different distribution patterns. Nevertheless, we also found that ORF52 is able to induce alterations in the MT cytoskeleton not only in hTERT-RhF but also in 293T cells (not shown), suggesting that the effects are not cell type specific. Of note, a recent report that appeared as we prepared the present study showed that KSHV ORF52 also associates with MTs (56). Although, in our hands, the effect from the overexpression of the KSHV ortholog is significantly less pronounced than that with RRV ORF52, this congruence suggests a likely conservation of function among gammaherpesviruses. Perhaps more remarkably, the tegument protein VP22 of the alphaherpesvirus HSV-1, despite sharing only low levels of regional sequence similarity with the gammaherpesvirus ORF52 orthologs, is a functional ortholog (32). Specifically, as with RRV ORF52, the ectopic expression of HSV-1 VP22 induces MT bundling and acetylation (45). Moreover, VP22 is essential for secondary envelopment during HSV-1 maturation, as is true for ORF52 in RRV (25) and MHV-68 (27). Finally, VP22 is responsible for the nuclear egress of other viral tegument proteins (57, 58), as we have shown for RRV (25).

A structure-based rationalization for the functional similarities has emerged from the X-ray crystallographic analysis of the core portion (residues 174 to 260) of HSV-1 VP22, which adopts a structure highly similar to the core portion (residues 44 to 106) of MHV-68 ORF52; their monomer α carbons align with a root mean square deviation of 2.1 Å (31, 32). Each monomer is comprised of a helix-loop-helix followed by a beta strand, which, together, form antiparallel homodimers. The similarity of the core structures of VP22 and MHV-68 ORF52 (and, by homology, ORF52 of RRV and other gammaherpesviruses) indicates that each protein could interact with common sets of cellular and viral proteins (32). Although other investigators have stated that VP22 fits the definition of a viral microtubule-associated protein (MAP) due to its ability to associate with and stabilize MTs (45), we are unaware of biochemical evidence that demonstrates that VP22 binds directly to tubulin *in vivo* or *in vitro*. Likewise, our data demonstrate that ORF52 colocalizes with and induces the formation of acetylated MT bundles. However, these findings do not preclude the possibility that ORF52 may recruit an intermediate, such as a cellular MAP, rather than binding directly to tubulin. We have also found that the changes in the MT cytoskeleton following ORF52 transfection or RRV infection show little resistance to either cold or the MT-depolymerizing agent nocodazole, suggesting that ORF52-induced MT cross-linking is likely not as robust as those mediated by some cellular MAPs or even those induced directly or indirectly by VP22 that resist depolymerization (45). Nevertheless, nocodazole added at 24 h p.i. markedly decreased (>4-fold) the amount of infectious RRV released into the medium over the subsequent 24 h (data not shown), suggesting that intact MTs are important, at a minimum, for overall virus production and/or egress.

The most striking structural domain that distinguishes gammaherpesvirus ORF52 orthologs from HSV-1 VP22 is the ORF52 N-terminal α1-helix. Notably absent in VP22, the ORF52 α1-helix contains four of the five residues (L27, E30, N31, and L34 for RRV ORF52) that are strictly conserved among gammaherpesvirus orthologs. (We discuss the fifth strictly conserved residue, R103, below.) Data from our transfection experiments indicate that three of these four α1-helix residues (L27, N31, and L34) are essential for robust MT bundling and acetylation as well as for the disruption of cytokinesis, while a mutation in the fourth residue (E30A) gives an intermediate phenotype. Sequence analysis and structural prediction software indicate that the α1-helices of nearly all the gammaherpesvirus ORF52 orthologs contain two heptad repeats that resemble a truncated leucine zipper with a potential for coiled-coil formation (35–38, 41). In addition, the length of the predicted coiled coil for each of these orthologs from a wide range of gammaherpesviruses (Table 1) varied minimally (3 to 6% for the Marcoil and PCOIL [window length of 14] algorithms, respectively), arguing for a conservation of structure-function for this motif (59). Our finding that the RRV ORF52 L20A mutation (as well as L27A and L34A) also leads to a null phenotype further buttresses the importance of the α1-helix heptad repeats in RRV ORF52-induced changes in MT architecture.

Clues into the mechanism underlying the role of the α1-helix leucine zipper motif in MT reorganization could lie within the crystal structure of MHV-68 ORF52 (31). The structure defines a homotetramer comprised of two antiparallel asymmetric dimers stabilized by a coiled coil involving the two most available α1-helices, one contributed from each dimer (31). We propose that the short length of each α1-helix would result in a weak coiled-coil interaction, thereby favoring a dynamic equilibrium between dimeric and tetrameric states (60). This dynamic could facilitate dimer dissociation and binding to different viral or cellular protein partners, including, in the setting of MT association, a cellular MAP. Potentially, mutation of any of the key residues of the α1-helix would disrupt the coiled coil and destabilize not only homotetramers but also ORF52-cellular protein complexes, providing a possible explanation for the loss of MT stabilization that we observe upon the mutation of any of the heptad leucine residues (61).

Additional evidence to support a role for the interactions of the ORF52 dimer with another cellular factor(s) during RRV-induced MT reorganization is our finding that the MT changes are at least partially dependent upon the strictly conserved β-strand Arg103 residue (positionally equivalent to Arg95 in MHV-68 and Arg242 in VP22). In both MHV-68 and VP22 dimers, the two arginines reside within an antiparallel β-sheet and contribute to an electrostatic potential surface map suggestive of protein-protein interactions (31, 32). For MHV-68, this residue is critical for ORF52-ORF42 complex formation (62). We propose that this strictly conserved residue, present in all gammaherpesvirus ORF52 orthologs as well as in VP22, might be a critical component for binding to one of a variety of different protein partners, which then modulates ORF52 (or VP22) function (25, 63, 64). For gammaherpesviruses, such combinatorial diversity could help explain the gamut of functions attributed to ORF52, including the nucleus-to-cytoplasm transport of other tegument proteins, virion secondary envelopment, anti-cyclic GMP-AMP synthase (cGAS) activity, and MT cytoskeletal changes (25, 27, 65).

In sum, we suggest a model in which ORF52 mediates marked effects on MT during both early and late stages of gammaherpesvirus infection and that this interaction depends on the formation of quaternary structures with a cellular factor(s). Although the potential advantages of stabilizing MTOC-associated MTs upon retrograde transport during initial gammaherpesvirus infection seem self-evident, the functions of extreme bundling, acetylation, and perinuclear MT wrapping during later stages of lytic infection are less clear. Possibilities include the MT-mediated sequestration of cellular factors that might otherwise serve to inhibit virion production. Others have found, for example, that MTs can bind to a proapoptotic factor, Bim, a member of the Bcl2 family (66), serving to prolong cell survival, and that a rabies virus-encoded MAP, P3, can bind to STAT1, inhibiting interferon (IFN)/STAT1-dependent signaling (1). Furthermore, MT bundling and acetylation mediated by increased ORF52 expression levels at late stages of infection could facilitate the translocation of maturing virions to different cellular compartments during nuclear egress, tegumentation, secondary envelopment, and vesicular fusion at the plasma membrane (67).

## MATERIALS AND METHODS

### Cell culture.

Telomerase-immortalized rhesus monkey fibroblasts (hTERT-RhFs) were grown in complete medium (Dulbecco's modified Eagle's medium [Life Technologies, Waltham, MA] supplemented with 1 nM puromycin, 1 mM sodium pyruvate, and 10% fetal bovine serum [Life Technologies]), as described previously (24).

### RRV stocks.

RhF cells were grown to confluence, at approximately 2 × 10^7^ cells, in a T182 flask and infected with RRV strain H26-95 at an MOI of 0.05 in 5 ml complete medium for 1 h. Cells were then supplemented with an additional 100 ml of complete medium per flask. Supernatants were collected at 5 days p.i. and cleared of cellular debris by low-speed centrifugation at 350 × *g* for 15 min at 4°C. Cleared viral supernatants were passed through a 0.45-μm-pore-size filter (EMD Millipore, Billerica, MA). Virus was concentrated by centrifugation for 3 h at 12,855 × *g* in a Sorvall SL250T rotor. The resulting viral pellets were resuspended on ice in 1.0 ml 1× phosphate-buffered saline (PBS).

### Antibodies.

Mouse monoclonal anti-RRV ORF52 and anti-RRV ORF65 (SCIP) were generated in the Lymphocyte Culture Center at the University of Virginia, as described previously (25). Anti-Infrared Dye 680 anti-mouse and anti-Infrared Dye 800 anti-rabbit were purchased from LiCor Biosciences (Lincoln, NE), rabbit polyclonal anti-acetyl-α-tubulin was obtained from Cell Signaling (Danvers, MA), mouse monoclonal anti-α-tubulin (clone DM1A) were obtained from Sigma-Aldrich (St. Louis, MO), rabbit polyclonal antipericentrin was obtained from Abcam (Cambridge, MA), and mouse monoclonal anti-c-Myc was obtained from Santa Cruz Biotechnology (Santa Cruz, CA). All Alexa Fluor secondary antibodies were purchased from Life Technologies.

### Plasmids.

ORF52 was cloned into the vector pK-Myc at the NotI and EcoRI sites of the multiple-cloning site. The full-length ORF52 sequence was amplified by PCR using purified RRV DNA as a template and then cloned into pK-Myc, as described previously (25). For the expression of ORF52 without a Myc tag, the pCMV-FLAG-C plasmid from Clontech (Mountain View, CA) was used. The full-length ORF52 sequence (which included the stop codon at the C terminus of ORF52) was amplified by PCR using the pK-Myc-ORF52 plasmid as a template and primers that added ApaI and KpnI sequences to ORF52 (ApaI-ORF52-F [5′-GCACGGGCCCAGATGTCTTCCACGCG-3′] and KpnI-ORF52-R [5′-GCAGGTACCCTAGTCCGCGTCGTTATTTC-3′]). RRV ORF52 was cloned into the ApaI and KpnI sites and sequenced to confirm that ORF52 was in frame, had the correct sequence, and contained the stop codon to prevent there from being a FLAG tag on the C terminus.

### RRV infections.

A total of 4 × 10^4^ cells/well were plated onto 48-well plates containing a Cell-Tak (BD Biosciences, San Jose, CA)-coated 8-mm-diameter coverslips (Electron Microscopy Sciences, Hatfield, PA) and incubated for 24 h. Cells were then infected with RRV at an MOI of 5 (or 50 for high-MOI experiments) for 1 h at 37°C with rocking every 15 min to ensure the uniform distribution of the virus. One hour later, the virus was removed, and the cells were washed with 1× PBS and then replaced with complete medium. Cells were then fixed at various times p.i.

### Immunofluorescence microscopy.

Cells were fixed with 4% formaldehyde in PHEM buffer [60 mM piperazine-*N*,*N*′-bis(2-ethanesulfonic acid) (PIPES), 25 mM HEPES, 10 mM EGTA, 2 mM MgCl_2_ (pH 6.9)] for 15 min at room temperature (RT). Cells were washed three times with PHEM buffer, permeabilized in 0.25% Triton in PHEM buffer for 10 min, and then washed three times with PHEM buffer. The samples were blocked overnight at 4°C in 10% normal goat serum (Jackson ImmunoResearch Laboratories, West Grove, PA) in PHEM buffer. Samples were stained at room temperature with antibodies diluted in 5% normal goat serum (in PHEM buffer). Primary antibodies were incubated for 1 h, and secondary antibodies were incubated for 30 min. Staining was sequential (i.e., the primary and then the corresponding secondary antibodies followed by the next primary antibody and its corresponding secondary antibody and so forth), with three washes with PHEM buffer between the addition of each antibody.

The following primary antibodies were used: anti-ORF52 (1:500), anti-acetyl-α-tubulin (1:250), antipericentrin (1:200), and anti-c-Myc (1:200). Secondary antibodies were either goat anti-mouse or anti-rabbit Alexa Fluor (Life Technologies, Waltham, MA). Anti-ORF52 and anti-c-Myc were detected with a goat anti-mouse secondary antibody conjugated to Alexa Fluor 568, while the antibodies to anti-acetyl-α-tubulin and pericentrin were detected with a goat anti-rabbit secondary antibody conjugated to Alexa Fluor 647. The antibodies to anti-α-tubulin and anti-ORF65 were conjugated directly to fluorophore 488 with a Mix-n-Stain CF488A kit (Biotium, Fremont, CA). After the last secondary antibody was added, and cells were counterstained with DAPI (4′,6-diamidino-2-phenylindole; Sigma-Aldrich) (1.0 μg/ml in double-distilled water) for 5 min at room temperature and washed once with double-distilled water. Coverslips were mounted onto microscope slides (Fisher) with Fluro-Gel (Electron Microscopy Sciences) and imaged with a Zeiss (Oberkochen, Germany) 710 confocal microscope.

### siRNA.

A Silencer Select custom siRNA specific for the RRV ORF52 coding sequence 5′-AACCCGTAAGATTGAAGCTAA-3′ and siControl 1 were purchased from Life Technologies.

### siRNA transfection followed by RRV infection.

A total of 20 nmol of ORF52 siRNA or 20 nmol of control siRNA was transfected into hTERT-RhF cells by using Lipofectamine RNAiMAX (Life Technologies) according to the manufacturer's protocol for reverse transfection. Sixteen hours later, cells were infected with RRV at an MOI of 5 for 1 h at 37°C with rocking every 15 min to ensure the uniform distribution of the virus. The medium containing the virus was then removed, and cells were washed with PBS and then incubated with complete medium for an additional 48 h. Cells were then either fixed for imaging or lysed for Western blot analysis (see below).

### ORF52 knockdown and rescue.

A total of 20 nmol of ORF52 siRNA or 20 nmol of control siRNA was transfected into hTERT-RhF cells by using Lipofectamine RNAiMAX (Life Technologies) according to the manufacturer's protocol for reverse transfection. Twenty-four hours later, cells were infected with RRV at an MOI of 5 for 1 h at 37°C with rocking every 15 min to ensure the uniform distribution of the virus. The medium containing the virus was then removed, and cells were washed with PBS and then incubated with complete medium for an additional 6 h. Cells (2 × 10^6^) were then transfected in suspension via Amaxa (Basel, Switzerland) Nucleofector program T-016 with 2 μg of the pK-Myc empty vector or the pK-Myc RRV ORF52 siRNA-resistant wild type. Cells were then plated onto 60-mm dishes. Cells were trypsinized from the plates 16 h after transfection and placed into 48-well plates containing Cell-Tak-treated 8-mm coverslips at a concentration of 1 × 10^5^ cells per well. Cells were incubated for another 24 h (total of 40 h posttransfection, 47 h postinfection, and 71 h post-siRNA transfection) before fixation.

### Protein electrophoresis and immunoblotting.

Cells were trypsinized off plates, pelleted, and washed once in 1× PBS. Pelleted cells were lysed for 10 min at 4°C with radioimmunoprecipitation assay (RIPA) buffer purchased from Sigma-Aldrich and supplemented with 1× protease inhibitor cocktail (Roche Life Science, Indianapolis, IN) immediately prior to use. Lysed cells were centrifuged for 10 min at 4°C, and supernatants were removed for protein analysis. Cleared cell lysates were resuspended in lithium dodecyl sulfate (LDS) sample buffer (NuPage; Thermo Fisher) with NuPage sample-reducing agent (50 mM dithiothreitol [DTT]). Following denaturation at 100°C for 5 min, proteins were separated by sodium dodecyl sulfate-polyacrylamide gel electrophoresis (SDS-PAGE) on 12% Bis-Tris gels (NuPage; Thermo Fisher).

For immunoblot analyses, proteins separated by SDS-PAGE were transferred onto nitrocellulose membranes for 60 min at 250 mA at 4°C. The membranes were blocked in 5% nonfat milk–TBS (20 mM Tris base, 150 mM NaCl, 3 mM Tris-HCl) overnight at 4°C and then incubated with primary antibodies in 5% nonfat milk in TBS-Tween (0.05%) for 90 min at RT. The following primary antibodies were used: anti-c-Myc (1:500), anti-acetyl-α-tubulin (1:500), anti-α-tubulin (1:500), and anti-RRV ORF52 (1:1,000). After three washes with TBS-Tween (0.05%) at RT, membranes were incubated with secondary antibodies for 45 min at RT. For quantitative immunoblotting, membranes were incubated with either Infrared Dye 800-conjugated anti-rabbit (1:5,000) or Infrared Dye 680-conjugated anti-mouse (LiCor Biosciences) diluted 1:10,000 in 5% nonfat milk in TBS-Tween (0.05%). Images were scanned and analyzed by using an Odyssey infrared CLx imaging system and Image Studio version 5.2 (LiCor Biosciences).

### Generation of an si52-resistant RRV ORF52 expression plasmid.

N-terminally Myc-tagged si52-resistant RRV ORF52 was generated by site-directed mutagenesis of pK-Myc-ORF52 as described previously (25).

### Generation of RRV ORF52 alanine mutations.

pK-Myc-ORF52 L27A, E30A, N31A, L34A, and R103A mutations were generated by site-directed mutagenesis using the QuikChange Lightning site-directed mutagenesis kit (Agilent Technologies, Santa Clara, CA). All mutations were confirmed by sequencing. The specific primers are as follows: ORF52 L27A Forward (5′-CAGGTGCAAAAAGCGTCCGTTGAAAAC-3′), ORF52 L27A Reverse (5′-GTTTTCAACGGACGCTTTTTGCACCTG-3′), ORF52 E30A Forward (5′-GCAAAAATTGTCCGTTGCAAACAAGCAGC-3′), ORF52 E30A Reverse (5′-GCTGCTTGTTTGCAACGGACAATTTTTGC-3′), ORF52 N31A Forward (5′-CAGGTGCAAAAATTGTCCGTTGAAGCCAAGCAGCTCAAAAAGCTG-3′), ORF52 N31A Reverse (5′-CAGCTTTTTGAGCTGCTTGGCTTCAACGGACAATTTTTGCACCTG-3′), ORF52 L34A Forward (5′-CCGTTGAAAACAAGCAGGCCAAAAAGCTGATAAATTCTGGG-3′), ORF52 L34A Reverse (5′-CCCAGAATTTATCAGCTTTTTGGCCTGCTTGTTTTCAACGG-3′), ORF52 R103A Forward (5′-GGAATTAGTATCGCCGTGGACGTGTC-3′), and ORF52 R103A Reverse (5′-GACACGTCCACGGCGATACTAATTCC-3′).

### Transfections.

hTERT-RhF cells (2 × 10^6^) were transfected in suspension via Amaxa (Basel, Switzerland) Nucleofector program T-016 with 2 μg of the pK-Myc empty vector, the pK-Myc RRV ORF52 siRNA-resistant wild type, or pK-Myc RRV ORF52 siRNA-resistant alanine mutants. Cells were then plated onto 60-mm dishes. For IF analysis, cells were trypsinized from the plates 24 h after transfection and placed into 48-well plates containing Cell-Tak-treated 8-mm coverslips at a concentration of 1 × 10^5^ cells per well. Cells were incubated for another 24 h (total of 48 h posttransfection) before fixation. For Western blot analyses, cells were allowed to remain on 60-mm dishes for 48 h before lysis.
